# Anti-sclerostin antibody and mechanical loading appear to influence metaphyseal bone independently in rats

**DOI:** 10.3109/17453674.2011.625539

**Published:** 2011-11-24

**Authors:** Fredrik Agholme, Hanna Isaksson, Xiaodong Li, Hua Zhu Ke, Per Aspenberg

**Affiliations:** ^1^Orthopaedics Division, Department of Clinical and Experimental Medicine, Faculty of Medicine, Linköping University, Linköping, Sweden; ^2^Department of Applied physics, University of Eastern Finland, Kuopio, Finland; ^3^Metabolic Disorders, Amgen Inc., Thousand Oaks, CA, USA

## Abstract

**Background and purpose:**

Sclerostin is produced by osteocytes and is an inhibitor of bone formation. Thus, inhibition of sclerostin by a monoclonal antibody increases bone formation and improves fracture repair. Sclerostin expression is upregulated in unloaded bone and is downregulated by loading. We wanted to determine whether an anti-sclerostin antibody would stimulate metaphyseal healing in unloaded bone in a rat model.

**Methods:**

10-week-old male rats (n = 48) were divided into 4 groups, with 12 in each. In 24 rats, the right hind limb was unloaded by paralyzing the calf and thigh muscles with an injection of botulinum toxin A (Botox). 3 days later, all the animals had a steel screw inserted into the right proximal tibia. Starting 3 days after screw insertion, either anti-sclerostin antibody (Scl-Ab) or saline was given twice weekly. The other 24 rats did not receive Botox injections and they were treated with Scl-Ab or saline to serve as normal-loaded controls. Screw pull-out force was measured 4 weeks after insertion, as an indicator of the regenerative response of bone to trauma.

**Results:**

Unloading reduced the pull-out force. Scl-Ab treatment increased the pull-out force, with or without unloading. The response to the antibody was similar in both groups, and no statistically significant relationship was found between unloading and antibody treatment. The cancellous bone at a distance from the screw showed changes in bone volume fraction that followed the same pattern as the pull-out force.

**Interpretation:**

Scl-Ab increases bone formation and screw fixation to a similar degree in loaded and unloaded bone.

The secreted glycoprotein sclerostin is the product of the SOST gene. Sclerostin is an important negative regulator of bone, and naturally occurring mutations of the SOST gene in humans lead to the high bone mass condition sclerostosis (Balemans et al. 2001, Brunkow et al. 2001). This high bone mass phenotype is also present in animal models of SOST deficiency (Li et al. 2008). Sclerostin asserts its function, in part, by inhibiting canonical Wnt signaling (Li et al. 2005). This is important for osteoblast differentiation (Galli et al. 2010) and also for bone healing and regeneration (Chen et al. 2007, Kim et al. 2007). The SOST gene is expressed almost exclusively in osteocytes (Poole et al. 2005), and sclerostin expression is thought to be a means for osteocytes to locally regulate bone formation (Galli et al. 2010). Sclerostin appears to be vital for the bone to be able to respond to mechanical loading (Robling et al. 2008), and lack of sclerostin prevents osteopenia due to unloading (Lin et al. 2009). One therapeutic option has been to block sclerostin with an antibody. Such treatment has increased bone mass in animal models of postmenopausal osteoporosis (Li et al. 2009) or disuse-induced bone loss (Tian et al. 2011), and in gonad-intact aged male rats and non-human primates (Li et al. 2010, Ominsky et al. 2010). Furthermore, fracture healing has been found to be improved in rodents and non-human primates treated with an anti-sclerostin antibody (Agholme et al. 2010, Ominsky et al. 2011).

We have previously shown that inhibition of sclerostin improves bone regeneration and implant fixation during normal loading conditions (Agholme et al. 2010). However, in contrast to laboratory animals, many patients do not bear weight on fractured limbs for a long time. It is therefore important to determine the effect of sclerostin inhibition on bone healing under unloaded conditions.

Paralysis of hind limb muscles using botulinum toxin A (Botox) causes rapid bone loss due to reduced weight bearing (Chappard et al. 2001, Warner et al. 2006). We examined the effect of sclerostin inhibition on metaphyseal bone healing in a rat model with Botox injections. Fully weight-bearing animals were included as controls.

## Materials and methods

Forty-eight 10-week-old male Sprague-Dawley rats (Taconic, Lille Skensved, Denmark) with a mean weight of 330 (SD 18) g were used. To unload the bone, 24 animals were injected with Botox (Allergan, Irvine, CA) in the extensor muscles of the right hind leg 3 days before surgery. All animals had a stainless steel screw inserted unilaterally in the right proximal tibia (Agholme et al. 2010). After surgery, the rats were randomly divided into 4 groups of 12 animals. One Botox-treated (unloaded) group and one untreated (loaded) group received subcutaneous injections of 25 mg/kg Scl-Ab twice weekly for 4 weeks, with injections starting 3 days after surgery. The other 2 groups were injected with saline solution at the same time points. The rats were killed 4 weeks after surgery.

### Implants

Stainless steel (316L) screws (thread M 1.7) were used. The threaded part of the screw is 2.8 mm long. The screws were custom-made and fitted with a head that enabled it to be mounted in a materials testing machine. The head has a 3.3-mm long portion that protrudes into the subcutaneous space. This type of screw has been used in this model previously (Agholme et al. 2010).

### Antibody

An anti-sclerostin monoclonal antibody (Scl-AbVI) specifically designed for rat studies was provided by Amgen Inc. (Thousand Oaks, CA).

### Botox injection

In animals allocated to Botox treatment, the right hind limb was shaved and cleaned with chlorhexidine alcohol. The rats were anesthetized with isoflurane and using an insulin syringe, they were given 5 × 1 U of Botox intramuscularly in the calf and quadriceps femoris muscles. 2 days after injection, none of the injected animals were using the injected limb. After 2 weeks, the injection procedure was repeated to ensure that the effect of the Botox did not recede.

### Surgical procedure

The rats were anesthetized with isoflurane and operated under sterile conditions. Each rat received 7 mg oxytetracycline and 0.015 mg buprenorphine at the time of surgery. The right hind limb was shaved and cleaned with chlorhexidine alcohol. The rat was placed in a sterile surgical glove and the shaved leg was pulled out through a hole in the glove. Sterile tape was wrapped around the paw, and the leg was cleaned once more with chlorhexidine alcohol. The medial proximal metaphysis was exposed with a longitudinal incision. The periosteum was reflected proximally up to the epiphysis. An insertion hole was hand-drilled in the cancellous bone, approximately 3 mm distal to the physis, using a regular 1.2-mm injection needle. A screw was inserted in the hole and screwed in place. The skin was sutured using a 4/0-monofilament nylon suture. The animals were allowed to bear full weight immediately after waking from anesthesia. They received 0.007 mg buprenorphine as postoperative analgesic every 12 h for 48 h. The rats were given free access to food and water during the experiment, and were housed 3 per cage at 21°C in a room with a cycle of 12 h light and 12 h dark. The study was approved by the Regional Ethics Committee for Animal Experiments and we followed institutional guidelines for care and treatment of laboratory animals.

### Mechanical evaluation

The rats were killed using carbon dioxide at the designated time point. Harvested bones were kept moist by saline irrigation and all bones were tested within 1 h of harvesting. All screws were tested for pull-out strength in a computerized materials testing machine (100 R; DDL Inc.), at a cross-head speed of 0.1 mm/s. The machine recorded the maximum force and the energy uptake until the force had dropped to 90% of maximum. The stiffness was then decided by the operator as the slope of the linear portion of the force/distance curve. The peak pull-out force was considered the primary variable. All analyses were performed while being blinded for treatment. 3 animals were excluded, 2 due to surgical mistakes and 1 because the tibia fractured at the screw during harvesting. All exclusions were done under blind conditions. The 3 animals that were excluded belonged to Botox-treated groups.

### Micro-CT (µCT)

**Figure 1.**
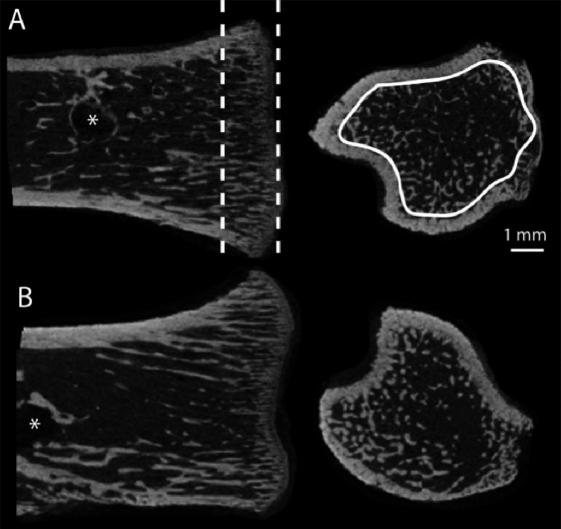
Region of interest used for µCT analysis. Frontal and transverse sections of the proximal tibia (epiphysis removed) from saline-treated (A) and Scl-Ab-treated (B) weight-bearing animals. A 1.5-mm long region in the metaphysis was chosen (dashed lines). In this region, only the trabecular bone was evaluated (white trace). This region was situated away from the screw (*) so that it would not be influenced by any bone formation around the screw.

After pull-out testing, the right proximal tibia of all animals was scanned using μCT (Skyscan 1172 v. 1.5; Skyscan, Aarteselar, Belgium). Figure 1 shows the region of interest analyzed. The µCT scanner acquired topographic images of the bone with an isotropic voxel size of 15 µm at energy settings of 100 kV and 100 µA, using aluminum filter of 0.5 mm and 10 repeated scans. The images were reconstructed using NRecon (Skyscan v. 1.5.1.4; Aarteselar), by correcting for ring artifacts and beam hardening. Analysis of bone volume fraction (BV/TV, when measured by µCT) and trabecular thickness, separation, and number (Tb.Th, Tb.Sp, Tb.N) were performed in CTAn (Skyscan v. 1.9.1.0; Aarteselar). BV/TV was considered the primary variable. All analyses were performed in a blinded manner. 4 additional samples had to be excluded since the µCT measurement region (specified in Figure 1) was too damaged to be properly evaluated. These exclusions were done while blinded. All animals that were excluded belonged to the Botox-treated groups.

### Statistics

Results are presented as mean (SD). Comparisons for group mean differences were calculated for treatment effects on log-transformed data, if variances were unequal—as decided by Levene's test. Differences were tested using 2-way ANOVA with Scl-Ab and Botox treatment as fixed factors, and t-test for post hoc comparisons. A result was considered to be statistically significant at p-values < 0.05. All statistical analyses were performed using SPSS v. 18.0.

## Results

The mean body weight of all groups increased during the experiment, but Botox-injected rats grew less. They weighted 22 (6) g less than uninjected rats at the time of surgery, and 75 (11) g less at the end of the experiment. The latter weight difference cannot be explained only by a reduced muscle mass in the treated hind limbs (data not shown).

### Implant fixation

2-way ANOVA confirmed an effect of both Scl-Ab and unloading on pull-out force (p < 0.001 for both), but we did not find any statistically significant interaction between them. Thus, the response to the antibody appeared to be similar in loaded and unloaded bone.

**Figure 2.**
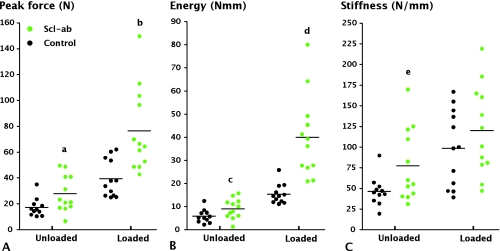
Effect of treatment with anti-sclerostin antibody (Scl-Ab) on screw fixation. A. Peak pull-out force. B. Pull-out energy. C. Stiffness. Compared to controls, Scl-Ab treatment significantly increased peak force (a: p = 0.01; b: p < 0.001) and energy (c: p = 0.02; d: p < 0.001) both with and without loading. Stiffness was significantly increased in an unloaded setting (e: p = 0.03). Unloading had a large effect on screw fixation, causing a large reduction in peak force (p < 0.001).

Unloading reduced the pull-out force by 56% compared to the controls with weight bearing (p < 0.001) (Figure 2). In unloaded bone, Scl-Ab treatment increased the pull-out force by 62% (p = 0.01) with corresponding increases in energy and stiffness (Figure 2). Antibody treatment did not restore the pull-out force (p = 0.05) or energy (p < 0.001) to values corresponding to those for weight-bearing controls. In weight-bearing bone, Scl-Ab treatment almost doubled the pull-out force (p = 0.003) and increased pull-out energy and stiffness (Figure 2).

### µCT analysis

**Figure 3.**
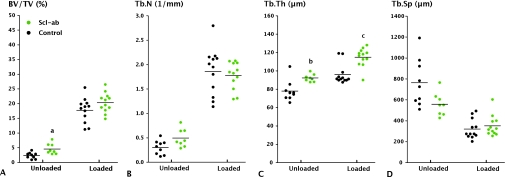
Effect of anti-sclerostin antibody (Scl-Ab) on trabecular bone volume in the proximal tibia, as measured by µCT. A. Bone volume fraction. B. Trabecular number. C. Trabecular Thickness. D. Trabecular separation. Botox treatment significantly reduced BV/TV, Tb.N, and Tb.Th compared to loaded controls (p < 0.001). Antibody treatment increased BV/TV both in the loaded and unloaded tibia, but only the increase in the unloaded tibia was significant (a: p = 0.03). Trabecular thickness was also significantly increased, however (b, c: p < 0.001).

2-way ANOVA confirmed an effect of both Scl-Ab (p = 0.029) and unloading (p < 0.001) on BV/TV (Figure 3). Both factors also influenced trabecular thickness (p < 0.001 for both). Unloading, but not antibody treatment, influenced trabecular number and trabecular spacing (p < 0.001 for both). However, there was no statistically significant interaction between unloading and antibody treatment for BV/TV or other morphometric parameters.

In unloaded controls, there was a 90% decrease in cancellous BV/TV compared to weight-bearing controls (p < 0.001). This decrease in BV/TV was primarily due to a profound decrease in trabecular number (p < 0.001). Trabecular thickness also decreased (p < 0.001), while trabecular spacing increased (p < 0.001).

Scl-Ab treatment of the unloaded bone increased BV/TV by 80% (p = 0.03). There was a 25% increase in trabecular thickness (p < 0.001) with no statistically significant change in trabecular number or spacing. In weight-bearing bone, Scl-Ab treatment caused a 20% increase in trabecular thickness (p < 0.001). There was no statistically significant increase in cancellous BV/TV (Figure 3), trabecular number, or spacing.

## Discussion

The quality of fixation of the implant may be considered to be the end result of a fracture-healing response of the kind that is prominent in cancellous bone. As in any stable metaphyseal fracture, this healing response leads to direct bone formation around the screw. This new bone is shaped like a thread, grasping the screw. The strength of this new bone, which reflects the fracture healing response, directly influences the force that will be needed to pull out the screw. We have previously shown that an increase in pull-out force using anti-sclerostin antibody is related to an increase in the amount of mineralized bone surrounding the screw (Agholme et al. 2010).

In the present study, Scl-Ab improved the fixation of a steel screw in the trabecular bone of normal-loaded and unloaded young male rats. Paralysis of muscles of the hind limb leads to rapid loss of bone mineral content and to changes in trabecular structure (Chappard et al. 2001, Warner et al. 2006). In this degraded bone, the Scl-Ab treatment had a positive effect. The raw data suggests that there was a greater response in loaded bone (Figure 2). However, for a correct statistical analysis, one requires log-transformation to equalize variation, and to compare the percentage changes rather than absolute differences. When this was performed, the interaction between loading and antibody disappeared.

Scl-Ab treatment increased BV/TV in the unloaded bone. These findings are consistent with a previous study in which administration of anti-sclerostin antibody prevented bone degradation after immobilization of the hind limb using bandages (Tian et al. 2011). In that study, Scl-Ab was shown to increase bone formation and reduce bone resorption in unloaded bone. In our study, the region of evaluation for μCT analysis included the primary spongiosa. This is different from other studies with Scl-Ab (Li et al. 2009, Tian et al. 2011), where the primary spongiosa was excluded. Nevertheless, Scl-Ab treatment induced an increase in trabecular thickness in both loaded and unloaded bone, with minimal effects on trabecular separation and number. This result is similar to that in a previous report on loaded bone (Agholme et al. 2010).

The expression of sclerostin is highly upregulated by unloading (Robling et al. 2008) and mice lacking sclerostin expression are insensitive to unloading (Lin et al. 2009). We had therefore expected a proportionately stronger response to the antibody treatment in unloaded bone. However, even with a high level of sclerostin inhibition, we could not fully counter the effects of unloading. This could have been due to very strong expression of sclerostin in the unloaded bone, in comparison to normally-loaded bone, in which case the amount of antibody administered may have been inadequate to fully inhibit sclerostin. It is also possible that sclerostin is not the only mechanically responsive modulator of bone during unloading. Expression of Dickkopf-1 (Dkk1), another Wnt inhibitor, is also upregulated during unloading. However, we recently showed that blocking of Dkk1 had no effect on pull-out strength parameters in unloaded bone despite the increase in pull-out strength parameters in normally-loaded bone (Agholme et al. 2011).

The present study had several limitations. We chose to only investigate the initial stages of fracture healing at a single time point. The study was designed to determine the effects of antibody treatment, with possible therapeutic applications in mind. To understand the physiological aspects, it would be more appropriate to study RNA and protein expression under different loading conditions. In addition, μCT evaluation in a region including the primary spongiosa might not reflect conditions in which growth plates are closed.

In summary, we found that the large anabolic effect of Scl-Ab on traumatized bone in young male rats can also be found in unloaded bone. Sclerostin inhibition could be a valuable therapeutic option in settings where the bone is unloaded. However, we were unable to find an interaction between Scl-Ab treatment and loading, suggesting that although sclerostin is expressed in the bone that is forming in response to metaphyseal trauma, this expression is not more sensitive to unloading than expression anywhere else.

## References

[CIT0001] Agholme F, Li X, Isaksson H, Ke HZ, Aspenberg P (2010). Sclerostin antibody treatment enhances metaphyseal bone healing in rats. J Bone Miner Res.

[CIT0002] Agholme F, Isaksson H, Kuhstoss S, Aspenberg P (2011). The effects of Dickkopf-1 antibody on metaphyseal bone and implant fixation under different loading conditions. Bone.

[CIT0003] Balemans W, Ebeling M, Patel N, Van Hul E, Olson P, Dioszegi M (2001). Increased bone density in sclerosteosis is due to the deficiency of a novel secreted protein (SOST). Hum Mol Genet.

[CIT0004] Brunkow ME, Gardner JC, Van Ness J, Paeper BW, Kovacevich BR, Proll S (2001). Bone dysplasia sclerosteosis results from loss of the SOST gene product, a novel cystine knot-containing protein. Am J Hum Genet.

[CIT0005] Chappard D, Chennebault A, Moreau M, Legrand E, Audran M, Basle MF (2001). Texture analysis of X-ray radiographs is a more reliable descriptor of bone loss than mineral content in a rat model of localized disuse induced by the Clostridium botulinum toxin. Bone.

[CIT0006] Chen Y, Whetstone HC, Lin AC, Nadesan P, Wei Q, Poon R (2007). Beta-catenin signaling plays a disparate role in different phases of fracture repair: implications for therapy to improve bone healing. PLoS Med.

[CIT0007] Galli C, Passeri G, Macaluso GM (2010). Osteocytes and WNT: the mechanical control of bone formation. J Dent Res.

[CIT0008] Kim JB, Leucht P, Lam K, Luppen C, Ten Berge D, Nusse R (2007). Bone regeneration is regulated by wnt signaling. J Bone Miner Res.

[CIT0009] Li X, Zhang Y, Kang H, Liu W, Liu P, Zhang J (2005). Sclerostin binds to LRP5/6 and antagonizes canonical Wnt signaling. J Biol Chem.

[CIT0010] Li X, Ominsky MS, Niu QT, Sun N, Daugherty B, D'Agostin D (2008). Targeted deletion of the sclerostin gene in mice results in increased bone formation and bone strength. J Bone Miner Res.

[CIT0011] Li X, Ominsky MS, Warmington KS, Morony S, Gong J, Cao J (2009). Sclerostin antibody treatment increases bone formation, bone mass, and bone strength in a rat model of postmenopausal osteoporosis. J Bone Miner Res.

[CIT0012] Li X, Warmington KS, Niu QT, Asuncion FJ, Barrero M, Grisanti M (2010). Inhibition of sclerostin by monoclonal antibody increases bone formation, bone mass, and bone strength in aged male rats. J Bone Miner Res.

[CIT0013] Lin C, Jiang X, Dai Z, Guo X, Weng T, Wang J (2009). Sclerostin mediates bone response to mechanical unloading through antagonizing Wnt/beta-catenin signaling. J Bone Miner Res.

[CIT0014] Ominsky MS, Vlasseros F, Jolette J, Smith SY, Stouch B, Doellgast G (2010). Two doses of sclerostin antibody in cynomolgus monkeys increases bone formation, bone mineral density, and bone strength. J Bone Miner Res.

[CIT0015] Ominsky MS, Li C, Li X, Tan HL, Lee E, Barrero M (2011). Inhibition of sclerostin by monoclonal antibody enhances bone healing and improves bone density and strength of non-fractured bones. J Bone Miner Res.

[CIT0016] Poole KE, van Bezooijen RL, Loveridge N, Hamersma H, Papapoulos SE, Lowik CW (2005). Sclerostin is a delayed secreted product of osteocytes that inhibits bone formation. FASEB J.

[CIT0017] Robling AG, Niziolek PJ, Baldridge LA, Condon KW, Allen MR, Alam I (2008). Mechanical stimulation of bone in vivo reduces osteocyte expression of Sost/sclerostin. J Biol Chem.

[CIT0018] Tian X, Jee WS, Li X, Paszty C, Ke HZ (2011). Sclerostin antibody increases bone mass by stimulating bone formation and inhibiting bone resorption in a hindlimb-immobilization rat model. Bone.

[CIT0019] Warner SE, Sanford DA, Becker BA, Bain SD, Srinivasan S, Gross TS (2006). Botox induced muscle paralysis rapidly degrades bone. Bone.

